# Multielement Analysis of Pakchoi (*Brassica rapa* L. ssp. *chinensis*) by ICP-MS and Their Classification according to Different Small Geographical Origins

**DOI:** 10.1155/2021/8860852

**Published:** 2021-02-09

**Authors:** Trung Nguyen-Quang, Giang Do-Hoang, Minh Truong-Ngoc

**Affiliations:** ^1^Vietnam Academy of Science and Technology (VAST), Center for Research and Technology Transfer (CRETECH), 18 Hoang Quoc Viet Road, 100000 Hanoi, Vietnam; ^2^Vietnam Academy of Science and Technology (VAST), Institute of Applied Mechanics and Informatics, 18 Hoang Quoc Viet Road, 100000 Hanoi, Vietnam

## Abstract

Statistical interpretation of the concentrations of 42 elements, determined using solution-based inductively coupled plasma mass spectrometry (ICP-MS) analysis and multivariate statistical methods, such as principal components analysis (PCA), was used to establish the provenance of pakchoi (*Brassica rapa* L. ssp. *chinensis*) from 6 areas in Ha Noi, Vietnam. Although pakchoi is widely cultivated and manufactured, no universal method is used to discriminate the origin of this vegetable. Our study introduced for the first time a method to classify pakchoi in small geographical areas. 42 metallic elements of pakchoi were detected by ICP-MS, which were further analyzed using multivariate statistical analysis to perform clusters based on the geographical locations. Eleven elements, i.e., ^28^Si; ^56^Fe; ^59^Co; ^63^Cu; ^69^Ga; ^75^As; ^85^Rb; ^93^Nb; ^107^Ag; ^118^Sn, and ^137^Ba, were identified as discriminators to distinguish pakchoi from those areas. Results from this study presented a new method to discriminant the geographical origins of pakchoi, which could apply to other types of vegetables on the food market.

## 1. Introduction

Discriminating the geographical origins of various food and drink products is growing considerably in recent years. In general, inductively coupled plasma mass spectrometry (ICP-MS) is one of the most useful approaches for multielement analysis of foods and drinks. It has been shown that ICP-MS data could be used successfully for discriminating wines from different geographical areas in Slovenia, Romania, and Croatia [1–3]. Chuzinska and Baralkiewicz determined 13 mineral elements for the classification of 55 honey samples in Poland [4]. Whereas, Chung et al. distinguished verified cultivation regions of potato by the stable isotope composition analysis of bioelements [5]. The geographical origin of vegetables, such as cabbages, was also discriminated by 21 inorganic elements through ICP-MS analysis [6]. The data of elements compositions are often combined with multivariate statistical analysis, such as principal component analysis (PCA), to reduce the dimension of the data matrix, visualize the similarities and differences among samples, and identify the key discriminants distinguishing the samples [7].

Pakchoi (*Brassica rapa* L.) is a major vegetable in Vietnamese diets. The consumption of pakchoi may provide many health benefits. Previous studies have shown that pakchoi potentially exerts inhibitory activity against cancer [8]. Phytochemical investigations have shown that flavonoids, hydroxycinnamic acids, and glucosinolate are the major bioactive ingredients of pakchoi, which possess high antioxidant activity [9, 10].

The chemical compositions of pakchoi are varied with cultivating conditions, such as soils, weather, and water resources [11, 12]. The growth and quality of pakchoi are significantly affected by inorganic elements, for example, selenate at low concentrations (0.5–1.0 mg/kg) could promote the growth of pakchoi and reduce cadmium contents in this vegetable [13]. The use of organic fertilizers and soil improvers, such as leonardite, might enrich the contents of macronutrients (Mg, Ca, K, and S) and micronutrients (Fe, Cu, Mn, and Zn).

The classification of different pakchoi cultivars has been conducted by different methods. Wiesner reported that pakchoi origin can be classified through their glucosinolate profile using HPLC-DA-MS analysis. In the glucosinolate profile, levels of 3-butenyl glucosinolate and 2-hydroxy-3-butenyl glucosinolate were considered important discriminators. By contrast, 5-methylsulfinylpentyl glucosinolate was found to be a characteristic ontogenetic variation of mature leaves of pakchoi by using PCA as the statistical model [14]. Another research also assessed the morphological and genetic diversity of pakchoi based on PCA and clustering analysis [15]. On the other hand, no previous approach categorized the geographical origins based on the characterization of elemental compositions of pakchoi. Thus, this study presents a promising method to discriminate against the geographical origins of pakchoi by using ICP-MS elemental analysis coupled with statistical principal component analysis. The proposed method may be utilized for discriminating against the origins of other vegetables.

## 2. Materials and Methods

### 2.1. Samples

**Figure 1:**
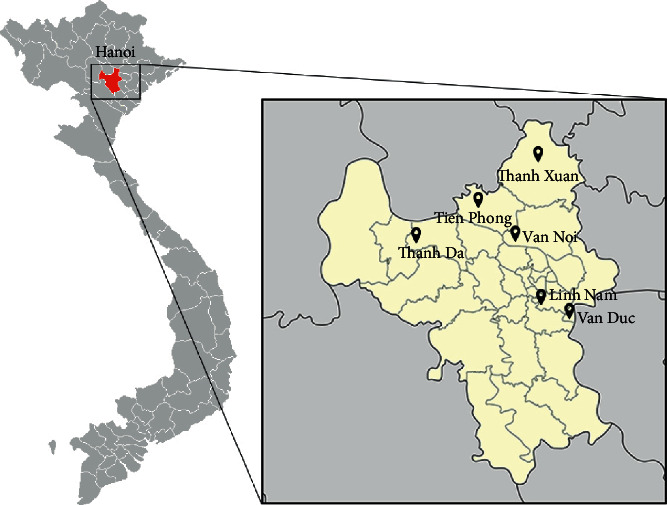
The geographical locations of the pakchoi cultivation regions.

Totally 60 pakchoi (*Brassica rapa* L. ssp*. chinensis*) samples were obtained from 6 different locations in Hanoi, Vietnam (Figure 1), which are Linh Nam (Hoang Mai district) 20°58′33.7″N 105°53′21.9″E, Tien Phong (Me Linh district) 21°09′27.2″N 105°44′19.4″E, Thanh Da (Phuc Tho district) 21°06′32.6″N 105°36′43.0″E, Thanh Xuan (Soc Son district) 21°14′04.6″N 105°45′59.3″E, Van Duc (Gia Lam district) 20°56′23.0″N 105°53′17.7″E, and Van Noi (Dong Anh district) 21°08′49.0″N 105°48′50.2″E. The samples were preliminarily sampled and prepared followed by Vietnam Standard TCVN 9016 : 2011 and TCVN 8551 : 2010 and then washed several times with distilled water.

### 2.2. Chemicals and Reagents

Nitric acid 65% (HNO_3_) and hydrogen peroxide 30% (H_2_O_2_) solutions were purchased from Merck, USA. Ultrapure deionized water with the resistivity of 18.2 MΩ cm was obtained from a Milli-Q Plus water puriﬁcation system (Millipore, Bedford, MA, USA). Twenty-six multielement standard solutions including ^11^B, ^23^Na, ^24^Mg, ^27^Al, ^28^Si, ^39^K, ^43^Ca, ^51^V, ^52^Cr, ^55^Mn, ^56^Fe, ^59^Co, ^60^Ni, ^63^Cu, ^66^Zn, ^69^Ga, ^75^As, ^85^Rb, ^88^Sr, ^107^Ag, ^111^Cd, ^121^Sb, ^133^Cs, ^137^Ba, ^202^Hg, and ^208^Pb (TraceCERT, periodic table mix 1 for ICP, product no. 92091, lot: BCBW5563) and nine rare earth elements (^45^Sc, ^89^Y, ^139^La, ^140^Ce, ^141^Pr, ^146^Nd, ^147^Sm, ^153^Eu, and ^157^Gd) 10 mg/L each element were provided by Sigma-Aldrich Company. A standard solution containing 50 *µ*g/L of ^47^Ti, ^90^Zr, ^93^Nb, ^95^Mo, ^105^Pd, ^118^Sn, and ^182^W in 6% ethanol/0.14 M HNO_3_ was used to determine the sensitivity factors for all elements across the entire mass range for the measurement of diluted samples made in the semiquantitative mode. In case of measuring digested samples, ethanol was omitted in the calibration solution. High-purity ethanol was used for preparing matrix-matched standards. Internal standards for quantitative analysis were prepared in 6% ethanol/0.14 M HNO_3_ for diluted samples and in 0.14 M HNO_3_ for digested samples. Blanks for the measurement of diluted and digested samples were a 6% ethanol/0.14 M HNO_3_ solution containing 50 *µ*g/L of the internal standard and a 0.14 M HNO_3_ procedure blank submitted to the microwave treatment and including the internal standard, respectively. Standards and the internal standard were prepared by appropriate dilution from 1000 mg/L standard stock solutions.

### 2.3. Sample Preparation and ICP-MS Measurements

All pakchoi samples were lyophilized at −45°C for 3 days and then ground with a pulverizer to obtain find powder (<400 *μ*m particle size) before storing in clean plastic bags. Samples (0.25 g) were weighed into a polytetrafluoroethylene (PTFE) vessel, and then, 4 mL H_2_O, 2 mL HNO_3_, and 2 mL H_2_O_2_ were added. After 30 min, the sample was digested in a Mars X-press plus microwave digestion system (CEM, NC, USA). The digestion program was as follows: the sample was heated to 12°C for 15 min, held for 10 min, before heating to 160°C for 10 min, held for 10 min, and finally heated to 180°C within 10 min and held for 30 min. After cooling, the solution was diluted to 25 mL in a volumetric flask with ultrapure water. The contents were then diluted with deionized water and analyzed [16]. An Agilent 7900 ICP-MS instrument (Agilent Technologies, Tokyo, Japan) was utilized for the measurement of 42 elements in the pakchoi samples, which were ^11^B, ^23^Na, ^24^Mg, ^27^Al, ^28^Si, ^39^K, ^43^Ca, ^45^Sc, ^47^Ti, ^51^V, ^52^Cr, ^55^Mn, ^56^Fe, ^59^Co, ^60^Ni, ^63^Cu, ^66^Zn, ^69^Ga, ^75^As, ^85^Rb, ^88^Sr, ^89^Y, ^90^Zr, ^93^Nb, ^95^Mo, ^105^Pd, ^107^Ag, ^111^Cd, ^118^Sn, ^121^Sb, ^133^Cs, ^137^Ba, ^139^La, ^140^Ce, ^141^Pr, ^146^Nd, ^147^Sm, ^153^Eu, ^157^Gd, ^182^W, ^202^Hg, and ^208^Pb. The analytical parameters of the ICP-MS were RF power at 1550 W, RF matching at 2.0 V, cell entrance at −40 V, cell exit of −60 V, cell energy discrimination at 5.0 V, spray chamber temperature at 2°C, argon was used as carrier gas at flowrate 1.09 L/min, and helium was used as auxiliary gas at 4.3 L/min. Data quantitation was achieved regarding matrix-matched multielement standards that had been prepared in 1% HNO_3_.

In this study, instrument detection limits were calculated using the raw intensity data from the standard and the blank (using ultrapure 2% nitric acid matrix) as per the following equation: IDL = 3SD_blank_*x C*_x_/(*S*_x_–S_blank_), where SD_blank_ is the standard deviation of the intensities of the multiple blank measurements, *C*_*x*_ is the mean signal for the standard, *S*_x_ is the signal for *C*_x_, and S_blank_ is the signal for blank. Method detection limits (MDLs) were calculated as follows: MDL = IDL *x* constant volume/sample weight.

Continuing calibration verification standards were prepared from single element ICP standards (Merck) consisting of 20 mg/L Ca and Mg for the high standard series, and 250 *µ*g/L Al, B, Cu, Rb, Sr, and Zn and 25 *µ*g/L Cd, Co, Cs, Ni, Tl, and V for the low standard series. The calibration verifications were measured after every 10 samples.

Duplicates of two pakchoi samples were prepared. Possible matrix effects were checked by running an interference check sample consisting of Ca (50 mg/L), Na (100 mg/L), Mg (150 mg/L), Fe (200 mg/L), Cu (250 mg/L), and Zn (500mg/L). In addition, spike recovery tests and serial dilutions were performed on pakchoi samples. The spiked samples were prepared at concentration levels of 20 and 100 *µ*g/L for the elements Al, Cu, and Sr and of 100 and 500 *µ*g/L for the elements B, Mn, Rb, and Zn. A serial dilution check (1 : 10 followed by 1 : 3, thus 1 : 30 final dilution) was performed on one pakchoi.

### 2.4. Statistical Analysis

The statistical analysis of the data was performed using STATISTICA 12 (Dell Software, United States). The principal component analysis (PCA) was applied to the data acquired from the ICP-MS analysis to evaluate the discriminants among 6 groups of pakchoi samples from 6 geographical locations. The outputs of multivariate statistical analysis included the scree plot to show contributions of principal components to the PCA model, the score scatterplot illustrates the separation of 6 different groups, the loading scatterplot explains the influences of the elements to the clustering, and the moving range charts illustrate the means and ranges of common distributions of variables on the cases.

## 3. Results and Discussion

### 3.1. Elemental Analysis of Pakchoi by ICP-MS

Table 1 shows the contents of 42 elements in pakchoi obtained from 6 areas in Hanoi, including Tien Phong, Thanh Da, Linh Nam, Thanh Xuan, Van Duc, and Van Noi. K was the most abundant element in pakchoi samples which accounted for 52–62% of the total elements. Furthermore, Ca was the second major element present in pakchoi which accounted for 30–40% of the total elements. The highest total element contents (about 21 mg/g of fresh material) were found in the samples from Van Duc and Thanh Xuan, whereas the samples from Linh Nam and Van Noi showed the lowest contents of elements at about 16 mg/g of fresh material. The concentration of each element in the samples varied significantly due to the differences in geographical locations.

### 3.2. Principal Components Analysis (PCA)

**Figure 2:**
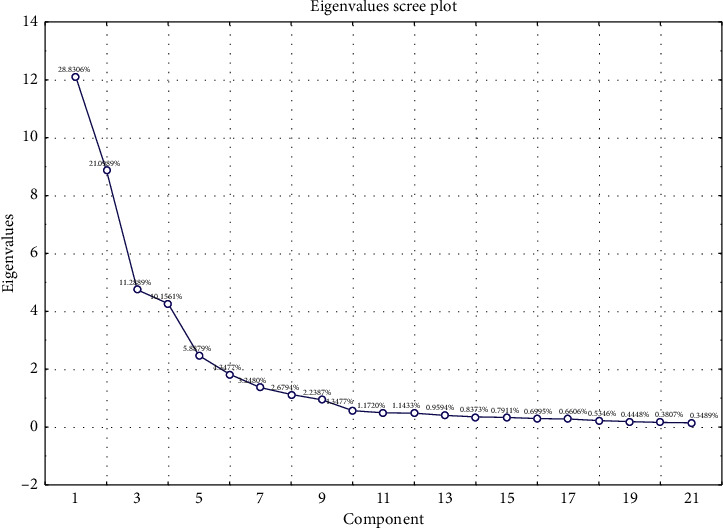
Eigenvalues scree plot of the principal components.

An unsupervised principal component analysis (PCA) was conducted to visualize the effects of geographical locations on elements and the discriminant of 60 pakchoi samples.  Figure 2 shows the scree plot which is used to illustrate the contributions of principal components (PCs) in a PCA model. As can be seen that the first three PCs accounted for more than 60% of the total variation of samples (Table 2), we can suppose that these PCs carried major information of variables. The quality of the PCA models was represented by *R*^2^ and Q^2^ values. The R^2^X_(cum)_ value at 0.499 and the Q^2^_(cum)_ value at 0.617 obtained from the PCA model were established by PC1 and PC2. The result indicated that 49.9% and 61.7% of the total variation could be explained and predicted, respectively, by the first two PCs.

**Figure 3:**
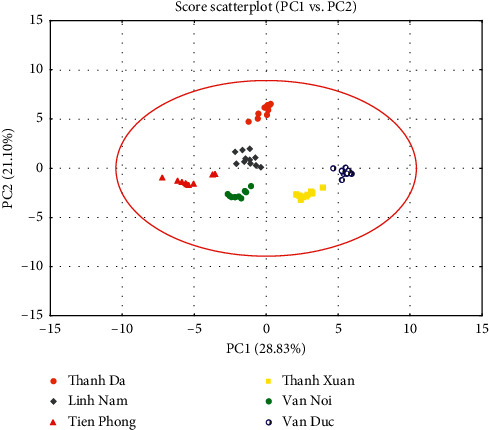
PCA scores scatterplot of principal components 1 and 2.

**Figure 4:**
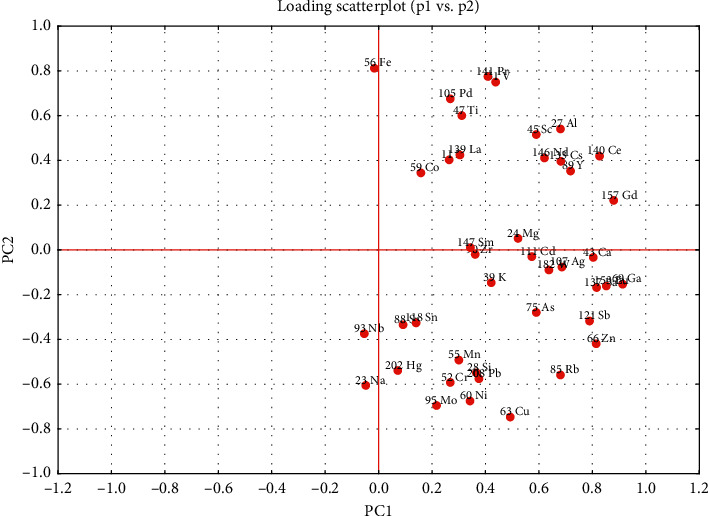
PCA loading scatterplots.

In the two-dimensional score scatterplot based on PC1 and PC2 (Figure 3), pakchoi samples from 6 areas were sharply separated, which highlighted the possibility to distinguish the origins of this vegetable merely by the metal elements distribution. In addition, the contribution of variables to classify was determined by the loading scatterplot (Figure 4). The contents of  ^69^Ga, ^157^Gd, and ^153^Eu had the highest weight on the first PC, while ^56^Fe and ^64^Cu showed the largest contribution to the separation on PC2.

**Figure 5:**
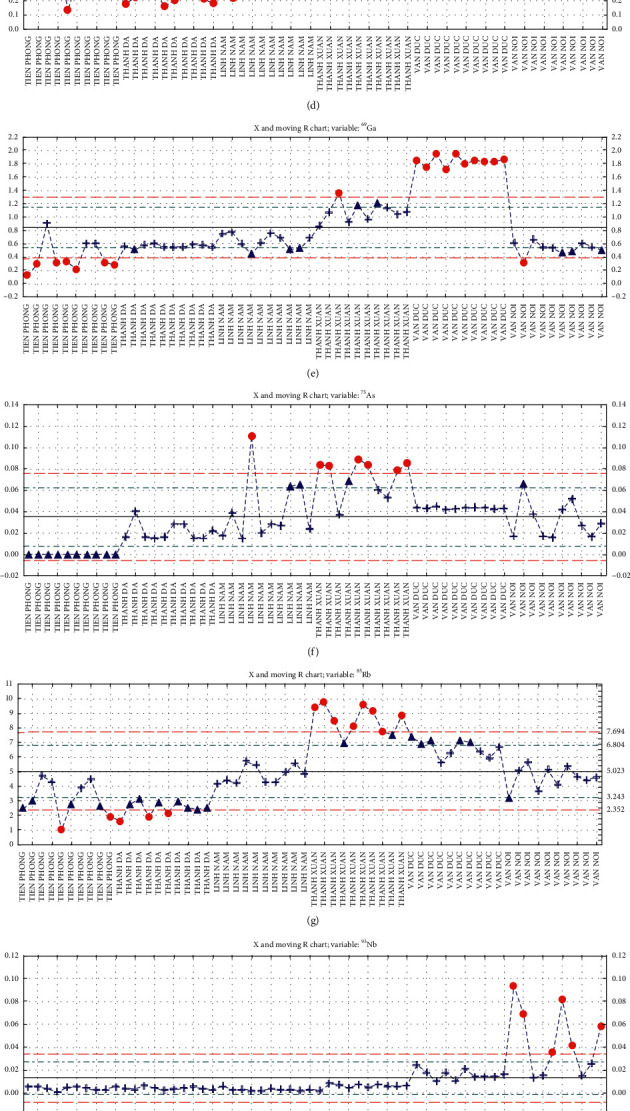
Moving range chart of (a) ^28^Si, (b) ^56^Fe, (c) ^59^Co, (d) ^63^Cu, (e) ^69^Ga, (f) ^75^As, (g) ^85^Rb, (h) ^93^Nb, (i) ^107^Ag, (j) ^118^Sn, and (k) ^137^Ba. The vertical axis illustrates the content (*µ*g/g, fresh weight base) of each element in the sample. The horizontal axis shows names of the areas.

Moving range charts illustrated the means and ranges of common distributions of variables on the cases. As can be seen, Thanh Da samples had significantly higher contents of ^56^Fe than the samples from other areas that could be used as a marker for distinguishing (Figure 5(b)). Meanwhile, in the samples from Linh Nam, the elemental isotopes ^59^Co and ^118^Sn were dramatically increased as compared to samples from other sites that could be found as two important discriminating elements for this area (Figures 5(c) and 5(j)). Whereas, a unique combination of three elements (^28^Si, ^63^Cu, and ^85^Rb) was identified as the main discriminator of pakchoi from Thanh Xuan (Figure 5(a), 5(d), and 5(g)). Similarly, a group of three elements, namely, ^69^Ga, ^107^Ag, and ^137^Ba, contributed strongly to the discrimination of samples from Van Duc (Figure 5(e), 5(i), and 5(k)). The levels of ^107^Ag and ^137^Ba increased sharply in Van Duc samples, compared to significantly low contents in the samples of other areas. For the Van Noi samples, the content of ^93^Nb was three-fold to tenth-fold higher than the levels of this element in the other samples (Figure 5(h)). Thus, ^93^Nb was responsible for the unique classification of samples from Van Noi. Interestingly, no elemental isotope was identified that contained at a dramatically high level in Tien Phong samples; however, it was possible to discriminate this area's samples based on the significantly low content of ^69^Ga, which was also identified as a discriminator for Van Duc (Figure 5(a)). This contrast was also seen on scores scatterplots where samples from Tien Phong and Van Duc distributed on two contrary ranges of PC1.

To the best of our knowledge, this is the first time the mineral elements are used for discrimination of pakchoi geographical origins. The chemometric-based ICP-MS approach was applied to distinguish many types of foods and plants, such as cabbage [6], chili [17], and tea [18]. PCA was used widely in those reports that could find clearly and preciously the most important elements for the classification of the analytes.

## 4. Conclusions

This study is the first report of the method to determine the geographical origin of various pakchoi samples based on ICP-MS analysis and multivariate statistical analysis (PCA). The results illustrated that eleven elemental discriminators for vegetables were identified, namely, ^28^Si, ^56^Fe, ^59^Co, ^63^Cu, ^69^Ga, ^75^As, ^85^Rb, ^93^Nb, ^107^Ag, ^118^Sn, and ^137^Ba. By using the PCA model, 60 samples of 6 areas in Hanoi were classified clearly with one-half of the variation that could be explained. The findings of this study imply that the discrimination of pakchoi based on their geographical locations could be well achieved by the combination of elemental profiling and multivariate statistical analysis. The reported method is convenient, fast, and environmentally friendly, with potential applications in distinguishing origins of a larger amount, ranges, and types of vegetables.

## Figures and Tables

**Table 1:** Contents (*µ*g/g, fresh weight base) of the 42 elements in pakchoi from 6 locations in Hanoi.

Element	LOD	LOQ	Cultivation region					
			Tien Phong	Thanh Da	Linh Nam	Thanh Xuan	Van Duc	Van Noi
			(*n* = 10)	(*n* = 10)	(*n* = 10)	(*n* = 10)	(*n* = 10)	(*n* = 10)
^11^B	0.031 ± 0.002	0.103 ± 0.006	<MQL	2.225 ± 0.001	2.296 ± 0.149	1.679 ± 0.254	1.103 ± 0.028	1.367 ± 0.129
^23^Na	2.220 ± 0.054	7.399 ± 0.179	740.7 ± 3.2	194.33 ± 3.43	343 ± 27	1056 ± 7	352.6 ± 3.6	401 ± 8
^24^Mg	9.090 ± 0.081	30.29 ± 0.269	528.8 ± 5.1	543.4 ± 5.9	536.5 ± 49.1	543.1 ± 42	629.6 ± 10.5	518.6 ± 6.7
^27^Al	1.100 ± 0.007	3.666 ± 0.023	58.85 ± 5.6	242.1 ± 7.7	130.5 ± 9.6	102.8 ± 1.2	305.8 ± 20.2	72.63 ± 4.16
^28^Si	28.07 ± 0.103	93.55 ± 0.342	45.77 ± 3.2	37.53 ± 1.83	212.4 ± 10.8	302.5 ± 33.1	126.7 ± 1.8	163.9 ± 9.9
^39^K	12.01 ± 0.051	40.02 ± 0.169	11369 ± 44	10464 ± 15	8683 ± 85	11915 ± 20	12113 ± 133	9220 ± 36
^43^Ca	4.003 ± 0.011	13.34 ± 0.036	5422 ± 41	6425 ± 62	6626 ± 61	7517 ± 56	8268 ± 52	5983 ± 24
^45^Sc	0.667 ± 0.004	2.223 ± 0.013	0.135 ± 0.011	0.272 ± 0.026	0.215 ± 0.121	0.213 ± 0.076	0.241 ± 0.012	0.207 ± 0.011
^47^Ti	0.429 ± 0.003	1.429 ± 0.009	1.383 ± 0.012	2.726 ± 0.121	1.246 ± 0.122	2.062 ± 0.169	1.608 ± 0.073	1.255 ± 0.191
^51^V	0.056 ± 0.006	0.186 ± 0.019	0.028 ± 0.002	0.065 ± 0.06	0.042 ± 0.003	0.034 ± 0.003	0.054 ± 0.002	0.031 ± 0.002
^52^Cr	0.072 ± 0.001	0.239 ± 0.003	0.123 ± 0.003	0.121 ± 0.007	0.111 ± 0.004	0.192 ± 0.001	0.157 ± 0.006	0.214 ± 0.006
^55^Mn	0.114 ± 0.002	0.379 ± 0.006	20.34 ± 1.07	9.976 ± 0.311	10.98 ± 0.11	22.9 ± 0.13	20.44 ± 0.18	14.11 ± 0.37
^56^Fe	2.030 ± 0.007	6.765 ± 0.023	20.39 ± 1.05	50.89 ± 0.18	31.59 ± 2.35	26.17 ± 2.21	21.12 ± 1.94	26.97 ± 0.43
^59^Co	0.005 ± 0.001	0.016 ± 0.003	0.018 ± 0.001	0.027 ± 0.002	0.041 ± 0.044	0.023 ± 0.002	0.026 ± 0.002	0.017 ± 0.001
^60^Ni	0.023 ± 0.003	0.076 ± 0.009	0.117 ± 0.001	0.065 ± 0.003	0.061 ± 0.005	0.279 ± 0.002	0.274 ± 0.092	0.364 ± 0.015
^63^Cu	0.027 ± 0.002	0.089 ± 0.006	0.35 ± 0.02	0.219 ± 0.011	0.441 ± 0.003	0.979 ± 0.019	0.702 ± 0.074	0.786 ± 0.036
^66^Zn	0.703 ± 0.026	2.343 ± 0.086	7.148 ± 0.09	9.204 ± 0.811	8.674 ± 0.572	15.73 ± 1.21	16.74 ± 0.302	12.73 ± 0.156
^69^Ga	0.070 ± 0.001	0.233 ± 0.003	0.399 ± 0.017	0.561 ± 0.005	0.636 ± 0.003	1.079 ± 0.025	1.835 ± 0.037	0.525 ± 0.035
^75^As	0.005 ± 0.001	0.016 ± 0.003	<MQL	0.022 ± 0.002	0.041 ± 0.002	0.073 ± 0.19	0.044 ± 0.004	0.032 ± 0.003
^85^Rb	0.004 ± 0.001	0.013 ± 0.003	3.111 ± 0.044	2.474 ± 0.218	4.783 ± 0.321	8.548 ± 0.581	6.638 ± 0.058	4.587 ± 0.139
^88^Sr	0.091 ± 0.016	0.303 ± 0.053	17.77 ± 0.32	15.57 ± 0.57	13.6 ± 1.2	18.5 ± 0.8	17.18 ± 0.01	15.81 ± 0.02
^89^Y	0.018 ± 0.001	0.059 ± 0.006	0.028 ± 0.004	0.046 ± 0.004	0.032 ± 0.001	0.044 ± 0.003	0.047 ± 0.002	0.0302 ± 0.0022
^90^Zr	0.0021 ± 0.0001	0.006 ± 0.0003	0.006 ± 0.001	0.004 ± 0.001	0.017 ± 0.001	0.0048 ± 0.0003	0.021 ± 0.001	0.0062 ± 0.0004
^93^Nb	0.0014 ± 0.0001	0.004 ± 0.0003	0.004 ± 0.001	0.004 ± 0.002	0.003 ± 0.003	0.0058 ± 0.004	0.016 ± 0.001	0.045 ± 0.002
^95^Mo	0.032 ± 0.002	0.106 ± 0.006	0.075 ± 0.003	0.032 ± 0.003	0.026 ± 0.002	0.155 ± 0.002	0.070 ± 0.031	0.093 ± 0.003
^105^Pd	0.0091 ± 0.0002	0.030 ± 0.0006	0.034 ± 0.002	0.056 ± 0.002	0.038 ± 0.003	0.039 ± 0.007	0.045 ± 0.003	0.035 ± 0.002
^107^Ag	0.0041 ± 0.0001	0.013 ± 0.0003	0.106 ± 0.007	0.005 ± 0.001	0.005 ± 0.001	0.042 ± 0.003	2.687 ± 0.449	0.018 ± 0.001
^111^Cd	0.0032 ± 0.0001	0.010 ± 0.0003	0.011 ± 0.001	0.023 ± 0.002	0.036 ± 0.003	0.027 ± 0.002	0.040 ± 0.003	0.033 ± 0.003
^118^Sn	0.061 ± 0.002	0.203 ± 0.006	0.093 ± 0.004	0.015 ± 0.001	0.255 ± 0.013	0.121 ± 0.001	0.155 ± 0.009	0.123 ± 0.007
^121^Sb	0.0035 ± 0.0001	0.011 ± 0.0003	0.042 ± 0.003	0.059 ± 0.004	0.041 ± 0.031	0.122 ± 0.005	0.131 ± 0.027	0.075 ± 0.005
^133^Cs	0.0027 ± 0.0002	0.008 ± 0.0006	0.012 ± 0.001	0.039 ± 0.003	0.041 ± 0.002	0.035 ± 0.003	0.044 ± 0.001	0.020 ± 0.001
^137^Ba	0.523 ± 0.048	1.743 ± 0.159	1.318 ± 0.094	1.31 ± 0.09	2.616 ± 0.111	2.763 ± 0.547	5.953 ± 0.479	1.64 ± 0.11
^139^La	0.009 ± 0.001	0.029 ± 0.003	0.016 ± 0.002	0.042 ± 0.002	0.044 ± 0.002	0.036 ± 0.002	0.023 ± 0.002	0.020 ± 0.002
^140^Ce	0.0052 ± 0.0001	0.017 ± 0.0003	0.018 ± 0.001	0.055 ± 0.002	0.031 ± 0.002	0.045 ± 0.002	0.057 ± 0.003	0.028 ± 0.007
^141^Pr	0.0017 ± 0.0001	0.005 ± 0.0003	0.003 ± 0.000	0.011 ± 0.001	0.0043 ± 0.0003	0.006 ± 0.001	0.0057 ± 0.002	0.0028 ± 0.0001
^146^Nd	0.010 ± 0.001	0.033 ± 0.003	0.012 ± 0.001	0.033 ± 0.002	0.015 ± 0.001	0.032 ± 0.013	0.024 ± 0.002	0.014 ± 0.001
^147^Sm	0.0025 ± 0.0001	0.008 ± 0.0003	<MQL	0.008 ± 0.001	0.008 ± 0.001	0.014 ± 0.001	0.0032 ± 0.0002	0.0053 ± 0.0003
^153^Eu	0.0028 ± 0.0002	0.009 ± 0.0006	0.003 ± 0.001	0.004 ± 0.001	0.006 ± 0.001	0.007 ± 0.498	0.014 ± 0.001	0.0038 ± 0.0001
^157^Gd	0.0014 ± 0.0002	0.004 ± 0.0006	0.003 ± 0.001	0.011 ± 0.001	0.006 ± 0.001	0.013 ± 0.001	0.015 ± 0.001	0.0038 ± 0.0002
^182^W	0.0011 ± 0.0001	0.003 ± 0.006	0.003 ± 0.001	0.002 ± 0.001	0.023 ± 0.002	0.014 ± 0.001	0.368 ± 0.007	0.012 ± 0.001
^202^Hg	0.0005 ± 0.0000	0.001 ± 0.0001	0.003 ± 0.001	0.0011 ± 0.0001	0.005 ± 0.001	0.0058 ± 0.0003	0.0045 ± 0.003	0.0072 ± 0.0001
^208^Pb	0.014 ± 0.001	0.046 ± 0.003	0.023 ± 0.002	0.0010 ± 0.0001	0.301 ± 0.002	0.489 ± 0.009	0.354 ± 0.002	0.512 ± 0.034

MQL, method quantification limit.

**Table 2:** Principal component analysis summary.

PC	R^2^X_(Cum)_	Q^2^X_(Cum)_	Eigenvalues
1	0.2883	0.2582	12.1088
2	0.4993	0.6172	8.8615
3	0.6122	0.8371	4.7413
4	0.7137	0.9498	4.2656
5	0.7726	0.9875	2.4729
6	0.8161	0.9975	1.8260
7	0.8486	0.9996	1.3642
8	0.8754	0.9999	1.1253
9	0.8978	1.0000	0.9402
10	0.9112	1.0000	0.5660

## Data Availability

The majority of the data used to support the findings of this study are included within the article. Other data are available from the corresponding author upon request.

## References

[1] Selih V. S., Sala M., Drgan V. (2014). Multi-element analysis of wines by ICP-MS and ICP-OES and their classification according to geographical origin in Slovenia. *Food Chemistry*.

[2] Geana I., Iordache A., Ionete R., Marinescu A., Ranca A., Culea M. (2013). Geographical origin identification of Romanian wines by ICP-MS elemental analysis. *Food Chemistry*.

[3] Krzlicova D., Fiket Z., Kniewald G. (2013). Classification of Croatian wine varieties using multivariate analysis of data obtained by high resolution ICP-MS analysis. *Food Research International*.

[4] Chudzinska M., Baralkiewicz D. (2010). Estimation of honey authenticity by multielements characteristics using inductively coupled plasma-mass spectrometry (ICP-MS) combined with chemometrics. *Food and Chemical Toxicology*.

[5] Chung I.-M., Kim J.-K., Jin Y.-I. (2016). Discriminative study of a potato (Solanum tuberosum L.) cultivation region by measuring the stable isotope ratios of bio-elements. *Food Chemistry*.

[6] Bong Y.-S., Song B.-Y., Gautam M. K., Jang C.-S., An H. J., Lee K.-S. (2013). Discrimination of the geographic origin of cabbages. *Food Control*.

[7] Low K. H., Zain S. M., Abas M. R. (2010). Evaluation of metal concentrations in red Tilapia *(Oreochromis spp)* from three sampling sites in jelebu, Malaysia using principal component analysis. *Food Analytical Methods*.

[8] Verhoeven D. T. H., Verhagen H., Goldbohm R. A., van den Brandt P. A., van Poppel G. (1997). A review of mechanisms underlying anticarcinogenicity by brassica vegetables. *Chemico-Biological Interactions*.

[9] Rochfort S. J., Imsic M., Jones R., Trenerry V. C., Tomkins B. (2006). Characterization of flavonol conjugates in immature leaves of pak choi [Brassica rapaL. Ssp.chinensisL. (hanelt.)] by HPLC-DAD and LC-MS/MS. *Journal of Agricultural and Food Chemistry*.

[10] Cartea M. E., Francisco M., Soengas P., Velasco P. (2011). Phenolic compounds in *Brassica* vegetables. *Molecules*.

[11] Adiloglu A., Eryilmaz Acikgoz F., Solmaz Y., Karaman M. R., Karaman M. R. (2018). The effects of the increased doses of leonardite applications on the content of some macro and micro nutrient elements of pak choi (Brassica rapa L. subsp. var. Chinensis L.) plant. *Eurasian Journal of Forest Science*.

[12] Pant A. P., Radovich T. J., Hue N. V., Talcott S. T., Krenek K. A. (2009). Vermicompost extracts influence growth, mineral nutrients, phytonutrients and antioxidant activity in pak choi (Brassica rapa cv. Bonsai, Chinensis group) grown under vermicompost and chemical fertiliser. *Journal of the Science of Food and Agriculture*.

[13] Li J., Liang D., Qin S., Feng P., Wu X. (2015). Effects of selenite and selenate application on growth and shoot selenium accumulation of pak choi (Brassica chinensis L.) during successive planting conditions. *Environmental Science and Pollution Research*.

[14] Wiesner M., Zrenner R., Krumbein A., Glatt H., Schreiner M. (2013). Genotypic variation of the glucosinolate profile in pak choi (Brassica rapa ssp.chinensis). *Journal of Agricultural and Food Chemistry*.

[15] Yu S., Zhang F., Wang X. (2010). Genetic diversity and marker-trait associations in a collection of Pak-choi (Brassica rapa L. ssp. chinensis Makino) Accessions. *Genes & Genomics*.

[16] Khan N., Jeong I. S., Hwang I. M. (2013). Method validation for simultaneous determination of chromium, molybdenum and selenium in infant formulas by ICP-OES and ICP-MS. *Food Chemistry*.

[17] Hwang I. M., Moon E. W., Lee H.-W. (2018). “Discrimination of chili powder origin using inductively coupled plasma–mass spectrometry (ICP-MS), inductively coupled plasma–optical emission spectroscopy (ICP-OES), and near infrared (NIR) spectroscopy. *Analytical Letters*.

[18] Liu H. l., Zeng Y. t., Zhao X., Tong H. r. (2020). Improved geographical origin discrimination for tea using ICP‐MS and ICP‐OES techniques in combination with chemometric approach. *Journal of the Science of Food and Agriculture*.

